# Diagnostic Features of Emotional Expressions Are Processed Preferentially

**DOI:** 10.1371/journal.pone.0041792

**Published:** 2012-07-25

**Authors:** Elisa Scheller, Christian Büchel, Matthias Gamer

**Affiliations:** 1 Department of Psychiatry and Psychotherapy, University Medical Center Freiburg, Freiburg, Germany; 2 Department of Systems Neuroscience, University Medical Center Hamburg-Eppendorf, Hamburg, Germany; University of California, Berkeley, United States of America

## Abstract

Diagnostic features of emotional expressions are differentially distributed across the face. The current study examined whether these diagnostic features are preferentially attended to even when they are irrelevant for the task at hand or when faces appear at different locations in the visual field. To this aim, fearful, happy and neutral faces were presented to healthy individuals in two experiments while measuring eye movements. In Experiment 1, participants had to accomplish an emotion classification, a gender discrimination or a passive viewing task. To differentiate fast, potentially reflexive, eye movements from a more elaborate scanning of faces, stimuli were either presented for 150 or 2000 ms. In Experiment 2, similar faces were presented at different spatial positions to rule out the possibility that eye movements only reflect a general bias for certain visual field locations. In both experiments, participants fixated the eye region much longer than any other region in the face. Furthermore, the eye region was attended to more pronouncedly when fearful or neutral faces were shown whereas more attention was directed toward the mouth of happy facial expressions. Since these results were similar across the other experimental manipulations, they indicate that diagnostic features of emotional expressions are preferentially processed irrespective of task demands and spatial locations. Saliency analyses revealed that a computational model of bottom-up visual attention could not explain these results. Furthermore, as these gaze preferences were evident very early after stimulus onset and occurred even when saccades did not allow for extracting further information from these stimuli, they may reflect a preattentive mechanism that automatically detects relevant facial features in the visual field and facilitates the orientation of attention towards them. This mechanism might crucially depend on amygdala functioning and it is potentially impaired in a number of clinical conditions such as autism or social anxiety disorders.

## Introduction

Human faces are stimuli we are exposed to every day. Throughout our lives, we have probably seen thousands of faces and certainly looked at some of them more closely to discover that they show various expressions. Human communication not only consists of voice messages but is disambiguated by gesture and facial expression. In line with this reasoning, emotionally expressive faces seem to be processed preferentially as compared to neutral ones [1], [2]. Thus, as social beings, it is important for us to be able to understand and interpret facially displayed emotions correctly. But how do we analyze faces to determine which expression they show us? The simple answer is that we seem to use diagnostic facial features. Already in 1944, Hanawalt showed that different facial features are important to distinguish between different specific emotions [3]. For example, he suggested the mouth to be most informative for recognizing happy faces and the eyes to be most important for detecting fearful facial expressions.

Recently, these findings were confirmed with the help of technically more sophisticated approaches. In 2001, the *Bubbles* technique was developed [4] and used to reveal that diagnostic features differ as a function of the task at hand [5]. This latter study revealed that the eye and mouth region across a wide range of spatial frequencies were diagnostic in an identity recognition task, which may indicate that the relationship between these features is crucial for the identification of a person. By contrast, the left side of the face around the eye region was most diagnostic for gender discrimination and the mouth region was most relevant for determining whether the depicted face showed a happy or neutral facial expression. Thus, it already appeared that different sets of facial features are diagnostic for different types of task. Using a similar technique, the distribution of diagnostic facial features was determined for each of the six basic emotional expressions [6]. In this study, observers yielded best results when the eye region was visible for fearful, the mouth region for happy and a mixture of these and other facial features for neutral facial stimuli.

An extraction of information from different facial features can also be assessed by monitoring eye movements and analyzing fixation patterns. Such a procedure was adopted by a number of studies focusing on clinical populations. For example, the comparison of visual scan paths of persons with autism spectrum disorder and a control group in an emotion classification task revealed a strong bias for primarily scanning the eye region in both groups [7]. Comparable results were reported by Hernandez and colleagues, who found a clear preference for fixating the eye region of sad, happy and neutral faces in autistic as well as in control subjects [8]. Additionally, there seemed to be a trend for spending relatively less time on the eye region and more time on the mouth of happy faces as compared to the other expressions in healthy controls. This may indicate that observers’ scanning behavior is sensitive to the diagnostic features of different emotional expressions. Although it is still debated whether patients with autism spectrum disorder scan (emotional) faces differently than healthy controls [9], [10], these studies reveal that eye tracking can be highly useful for elucidating information extraction processes during face perception.

However, a major drawback of these studies is the use of comparably long exposure times (typically longer than 2 s) which only allow for characterizing explicit face perception mechanisms which presumably are under conscious control. Recent evidence suggests that briefly presented faces also trigger very early, potentially reflexive, eye movements that are sensitive to the distribution of diagnostic facial features [11]. In this particular study, fearful, happy, angry and neutral faces were presented briefly (150 ms) so that observers were only able to accomplish a saccade after stimulus offset. Furthermore, the initial fixation was manipulated and subjects fixated on either the eye or mouth region in one half of all trials, respectively. Surprisingly, although saccades did not allow for extracting further information from the stimuli, observers showed a relatively large amount of fixation changes after stimulus offset. Across all facial expressions, reflexive gaze changes toward the eye region occurred much more frequently than fixation changes leaving the eye region. This is consistent with previous findings documenting a clear preference for using information from the eye region already at an early point in the time [12]. However, reflexive eye movements after stimulus offset were also sensitive to diagnostically relevant regions in the face [11]. Thus, gaze preferences for the eye region were largest for fearful and neutral faces and substantially reduced for happy facial expressions that triggered more fixation changes toward the mouth.

These findings indicate that human observers exhibit a tendency of automatically extracting information from the eye region of conspecifics. Additionally, they seem to be prone to quickly search for salient facial features that allow for validly identifying the current emotional state of the opponent. However, as most above-mentioned studies explicitly required participants to recognize the depicted emotional expression, it is unclear whether the observed gazing pattern reflects an automatic mechanism or is driven by task demands. Furthermore, previous studies did not examine whether eye movements were only modulated by low-level image features that trigger bottom-up attentional processes [13], [14] or reflect the influence of a top-down mechanism. To clarify these issues, we carried out two eye tracking studies. In Experiment 1, observers had to accomplish an emotion classification, a gender discrimination and a passive viewing (oddball) task using negative (fearful), positive (happy) and neutral facial expressions. To differentiate between early, potentially reflexive, eye movements on the one hand and a more elaborate scanning on the other, faces were either presented briefly (150 ms) or long enough to accomplish several saccades during stimulus presentation (2000 ms). In this experiment, we manipulated whether participants initially fixated on the eye or mouth region and we determined whether saccades and fixations showed a preference for the eye region in general [12] and for the varying diagnostic features of the emotional expressions [6], [11]. To rule out that low level saliency in certain parts of the images was driving these responses and to examine the general influence of the presentation position in the visual field, Experiment 2 was carried out using a specifically tailored set of stimuli with comparable saliency in the eye and mouth region across different emotional facial expressions. These faces were either presented in the upper half, the middle or the lower half of the display screen.

## Experiment 1

### Materials and Methods

#### Participants

This study was approved by the ethics committee of the medical faculty of the University of Rostock and conducted according to the principles expressed in the Declaration of Helsinki. All participants gave written informed consent and were paid for participation. Twenty-five students participated voluntarily in the experiment. One male subject was excluded because of too many invalid eye tracking trials. The final sample consisted of 12 women and 12 men, aged between 19 and 27 years (*M* = 24.13; *SD* = 3.62). All had normal or corrected to normal vision and were informed beforehand to wear contact lenses instead of glasses and to refrain from using eye make-up.

#### Design

The experiment was based on a 3 × 2 × 3 × 2 within-subjects design with the factors task, presentation time, emotional expression and initial fixation. These factors were specified as follows: 1) Subjects had to accomplish three different tasks in separate experimental blocks while eye tracking data was recorded: An emotion classification, a gender discrimination and a (passive) target detection task. 2) Within each block, portrait pictures of faces were either presented for 150 or 2000 ms and 3) these faces either showed fearful, happy, or neutral expressions. 4) Additionally, the initial fixation was systematically varied by unpredictably shifting faces down- or upwards on each trial such that subjects initially fixated either on the eye or mouth region.

#### Stimuli and tasks

Stimuli were presented on a 20″ Samsung SyncMaster 204B display (40.64 cm × 30.48 cm) with a resolution of 1600 by 1200 and a refresh rate of 60 Hz. The distance from the participants’ eyes to the monitor was 58 cm. The fearful, happy and neutral faces that were shown during the experiment were selected from several picture sets (The Karolinska directed emotional faces, KDEF, [15]; Pictures of facial affect, [16]; NimStim, www.macbrain.org/resources.htm; and the FACES database, [17]). These faces were slightly rotated such that both pupils were always on the same imagined horizontal line. Subsequently, pictures were converted to grayscale images and cropped with an ellipse in order to hide features that do not carry information on the emotional status of the conspecific (e.g. hair, ears). Finally, cumulative brightness was normalized across pictures. Overall, the pool of stimuli consisted of 126 fearful, 144 happy, and 140 neutral facial expressions. To control for the initial fixation, the stimuli within each emotional expression were shifted either downward or upward on each trial. This resulted in either the eye or the mouth region appearing at the location of the fixation cross.

**Figure 1:**
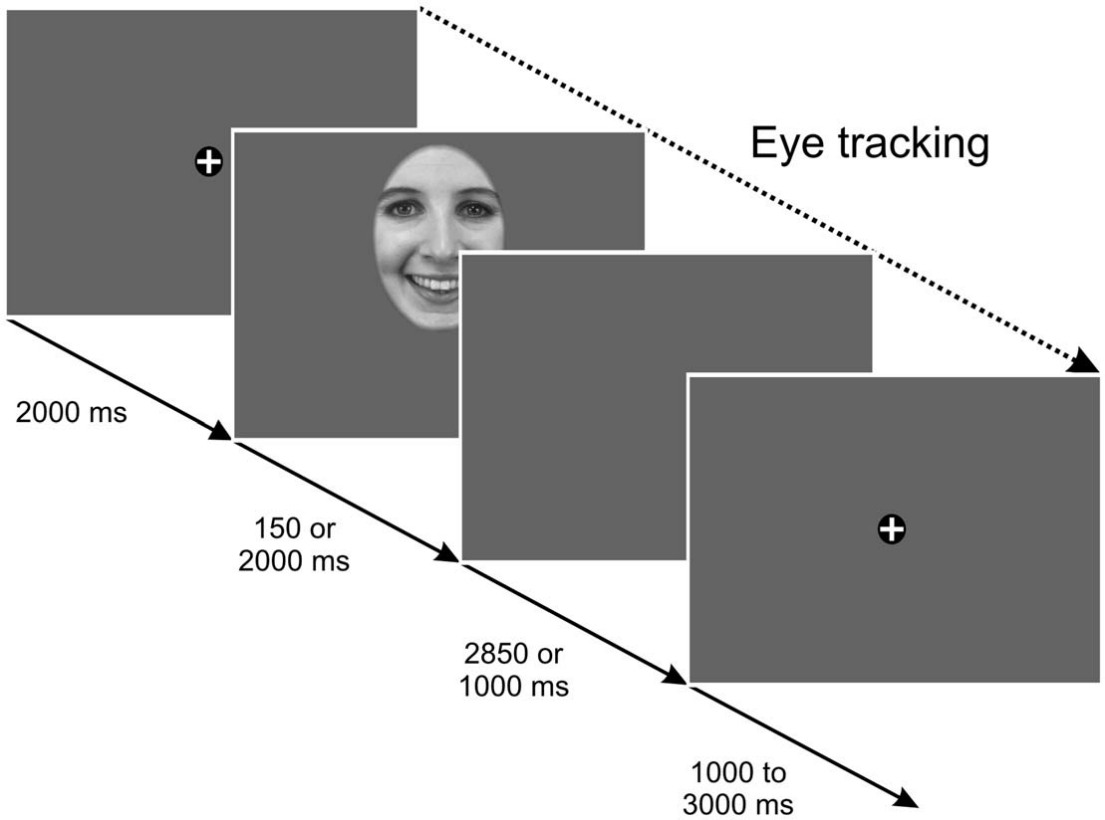
Illustration of the trial structure (Experiment 1).

Each trial began with a fixation cross shown for 2000 ms on a uniform grey background. Afterwards, faces were presented either for 150 or 2000 ms. The short duration was chosen to ensure that subjects could reliably identify the facial expression without being able to change their fixation during stimulus presentation. Any “reflexive” saccade that is related to the stimulus presentation would start when the picture already disappeared from the screen (for a similar procedure see [11], [18]). By contrast, within the larger presentation time of 2000 ms, subjects are able to accomplish several saccades during stimulus presentation to visually scan the picture in detail. To achieve an overall trial length of 5000 ms, a blank screen following picture presentation was either presented for 2850 or 1000 ms depending on whether the preceding picture was shown for the short or the long period. The period between two successive trials was varied randomly between 1000 and 3000 ms (see Figure 1). Presentation 13.0 (Neurobehavioral Systems) was used to control stimulus presentation and to record behavioral responses during the tasks.

In each experimental task, 36 pictures of different male and female persons were presented in a randomized order. The pictures were selected randomly for each subject from the whole pool of stimuli. Overall, males (*M* = 51.3%, *SD* = 2.7%) and females (*M* = 48.6%, *SD* = 2.7%) were presented approximately equally often. Each picture was shown twice and subjects had to accomplish different tasks as accurately and as quickly as possible by pressing the corresponding key on the computer keyboard. In the emotion classification task, subjects had to decide whether the face showed a fearful, happy or neutral facial expression. In the gender discrimination task, subjects were required to decide whether the displayed person was male or female. The target detection task was more or less passive with respect to the evaluation of the face. Subjects simply had to press one button whenever a rarely presented color image was shown. To this aim, eight color images (two males and two females with neutral facial expression) were randomly interspersed into the stimulus sequence.

Eye tracking data were recorded during the three tasks with a sampling rate of 1000 Hz using a Table-mounted Eyelink 1000 remote infrared pupil-corneal reflection eye imaging system (SR Research Ltd., Ottawa, Canada).

#### Procedure

After arriving at the laboratory, subjects completed a brief questionnaire asking for sociodemographic data (age, gender, profession, current medication). Afterwards, participants were verbally instructed before the eye tracking tasks started. In general, they were told to look at the screen during the experiment and to avoid large head and body movements. Whenever a fixation cross was presented during the tasks, they were told to fixate it continuously. While a picture was shown or when the screen was uniformly gray, they should feel free to change their gaze and look everywhere on the screen they wanted to. However, blinks during that period should be avoided. The order of the three tasks involving eye tracking was counterbalanced across participants. Each task started with 12 training trials (+2 color images in the passive condition) using a set of different faces. Subsequently, the eye tracking system was calibrated using nine points, the calibration was validated and the actual task started.

#### Data reduction and analysis

From the behavioral data, we calculated the proportion of hits (correct reactions) for each of the three eye tracking tasks. For the emotion classification- and the gender discrimination tasks, we examined effects of the experimental manipulations on the behavioral data using a 2 × 3 × 2 repeated measures ANOVA with the factors presentation time, emotion and initial fixation.

Two different measures were extracted from the eye tracking data: First, we analyzed the first saccade that was accomplished after stimulus onset. To this aim, we eliminated trials containing blinks and trials with saccades >1° occurring within a period of −300 to 150 ms relative to stimulus onset. Subsequently, we subtracted the prestimulus baseline from the position data of each valid trial to remove drifts. Afterwards, the first saccade exceeding 1° within 1000 ms after stimulus onset was detected. Furthermore, the saccade was required to occur at least 150 ms after stimulus onset (i.e. after the stimulus offset of the briefly presented faces). This saccade was classified according to whether it was directed towards the other major facial feature. Thus, when the eyes were presented at fixation, we identified the number of downward fixation changes toward the mouth and when the mouth followed the fixation cross, we calculated the corresponding proportion of upward saccades toward the eyes. These numbers were divided by the total number of valid trials to obtain proportions of fixation changes as a function of the experimental manipulations. Using a 3 × 2 × 3 × 2 repeated measures ANOVA on these proportions we tested for effects of task, presentation time, emotion and initial fixation on these “reflexive” saccades.

Second, fixation durations were analyzed for the 2000 ms stimulus duration. Valid trials were identified similarly to the saccadic data as described above and for these trials, we determined the amount of time subjects spent looking at either the eye region or the mouth region using predefined rectangular regions of interest that were centered on the respective facial feature. This cumulative fixation time on these regions was divided by the amount of time subjects spent looking at the presented face in general. To determine whether the experimental manipulations affected fixations on the eye and mouth region, we calculated a 3 × 3 × 2 × 2 repeated measures ANOVA on these proportions using the factors task, emotion, initial fixation and the facial feature that was actually fixated.

**Figure 2:**
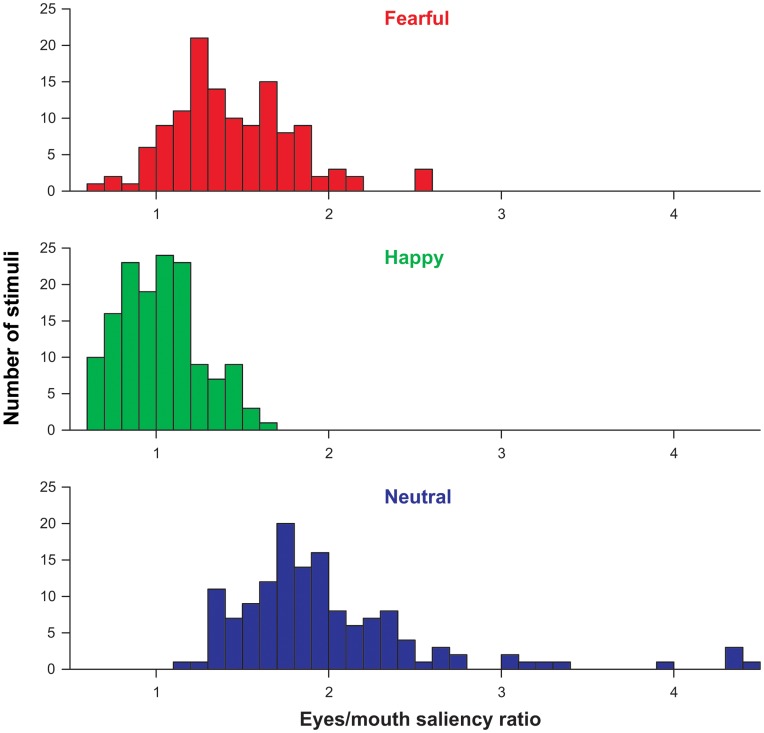
Frequency histograms showing the distribution of saliency ratios (average saliency in the eye region divided by the average saliency in the mouth region) for fearful, happy and neutral facial expressions.

Since it is possible that differential attention toward the eye or mouth region of the presented faces is driven by differences in low-level image features, we accomplished a second, post-hoc analysis taking into account the distribution of saliency across the face. To this aim, saliency maps were calculated for all faces that were used in the experiment. By using a biologically plausible processing hierarchy [14], the algorithm analyzes, for every pixel, how distinct that location on the image was along dimensions of luminance, contrast, orientation and spatial scale [13], [19]. The analysis results in a map for every stimulus used in the experiment showing image locations that stand out in terms of their low-level features from the background. For these maps, we computed the mean saliency in the eye or mouth region, respectively, using the same regions of interest as for the analysis of the fixation data. Finally, we calculated a saliency ratio by dividing the mean saliency of the eye region by that of the mouth region. As can be seen by the histogram depicted in Figure 2, the eye region had a generally higher saliency than the mouth region (ratios are above 1 on average). Furthermore, the distribution of saliency ratios differed between the emotional expressions. To ensure that the latter difference did not affect our results regarding the saccadic and the fixation data, we selected for each participant a subset of 6 pictures for each emotional expression, respectively, that had a similar saliency ratio across facial expressions. Mean ratios were 1.40 (*SD* = 0.05) for fearful, 1.39 (*SD* = 0.06) for happy, and 1.41 (*SD* = 0.05) for neutral facial expressions. These values did not differ significantly, *F*_(2,46)_ = 2.30, ε = .58, *p* = .14, partial η^2^ = .09. In addition to the analyses of the whole stimulus set, we calculated comparable repeated measures ANOVAs on the saccadic and the fixation data for this subset of faces. Due to the small amount of stimuli that had equal saliency ratios, we collapsed data across tasks and presentation times for these analyses.

For all repeated-measures ANOVAs involving more than one degree of freedom in the enumerator, the Huynh-Feldt procedure was applied to correct for potential violations of the sphericity assumption. A rejection criterion of *p*<.05 was used for all statistical tests and partial η^2^ is reported as an effect size index.

### Results and Discussion

#### Behavioral data

Overall, participants were very accurate in all three tasks. In the emotion classification task, mean proportions of correct responses were above 93% in all experimental conditions (see Table 1) and the statistical analysis did not reveal any significant effect of the experimental manipulations on the hit rates in this task.

**Table 1:** Experiment 1: Proportions of correct responses in the emotion classification and the gender discrimination task as a function of presentation time, initial fixation and emotional expression.

Presentation time	Initial fixation	Emotion		
		Fearful	Happy	Neutral
*Emotion classification task*				
150 ms	Eyes	97.2% (1.6%)	97.9% (1.1%)	97.2% (1.3%)
	Mouth	97.2% (1.3%)	93.1% (2.4%)	98.6% (1.0%)
2000 ms	Eyes	95.8% (1.8%)	96.5% (1.7%)	97.9% (2.1%)
	Mouth	97.2% (1.3%)	98.6% (1.0%)	98.6% (1.0%)
*Gender discrimination task*				
150 ms	Eyes	92.4% (3.0%)	95.1% (1.9%)	94.4% (2.8%)
	Mouth	82.6% (3.8%)	97.2% (2.2%)	94.4% (1.9%)
2000 ms	Eyes	95.1% (1.9%)	97.9% (1.1%)	98.6% (1.0%)
	Mouth	93.7% (2.0%)	97.2% (2.2%)	97.9% (1.1%)

*Note*. Standard errors of the mean are printed in brackets.

For the gender discrimination task, hit rates were similarly high (Table 1) but we observed larger hit rates when faces were visible for 2000 ms (main effect presentation time: *F*_(1,23)_ = 13.35, *p*<.001, partial η^2^ = .37). Furthermore, gender was identified more accurately for happy as compared to neutral and fearful faces (main effect emotion: *F*_(2,46)_ = 11.89, ε = 1.00, *p*<.001, partial η^2^ = .34). The main effect of the initial fixation as well as all interactions did not reach statistical significance.

In the passive viewing task, subjects reached an overall hit rate of 99.8% in detecting a non-target which means that they were almost always correct in not delivering a keypress when a monochrome stimulus was presented. The hit rate of target detections (i.e. keypresses for colored faces) was 100%.

#### Eye tracking data: First saccade after stimulus onset

The average number of valid trials without blinks or fixation changes between −300 and 150 ms relative to stimulus onset was 65.96 of 72 (*SD* = 5.03) in the emotion classification task, 67.21 of 72 (*SD* = 4.67) in the gender discrimination task and 68.33 of 72 (*SD* = 4.46, only trials with non-target stimuli) in the passive viewing condition.

**Figure 3:**
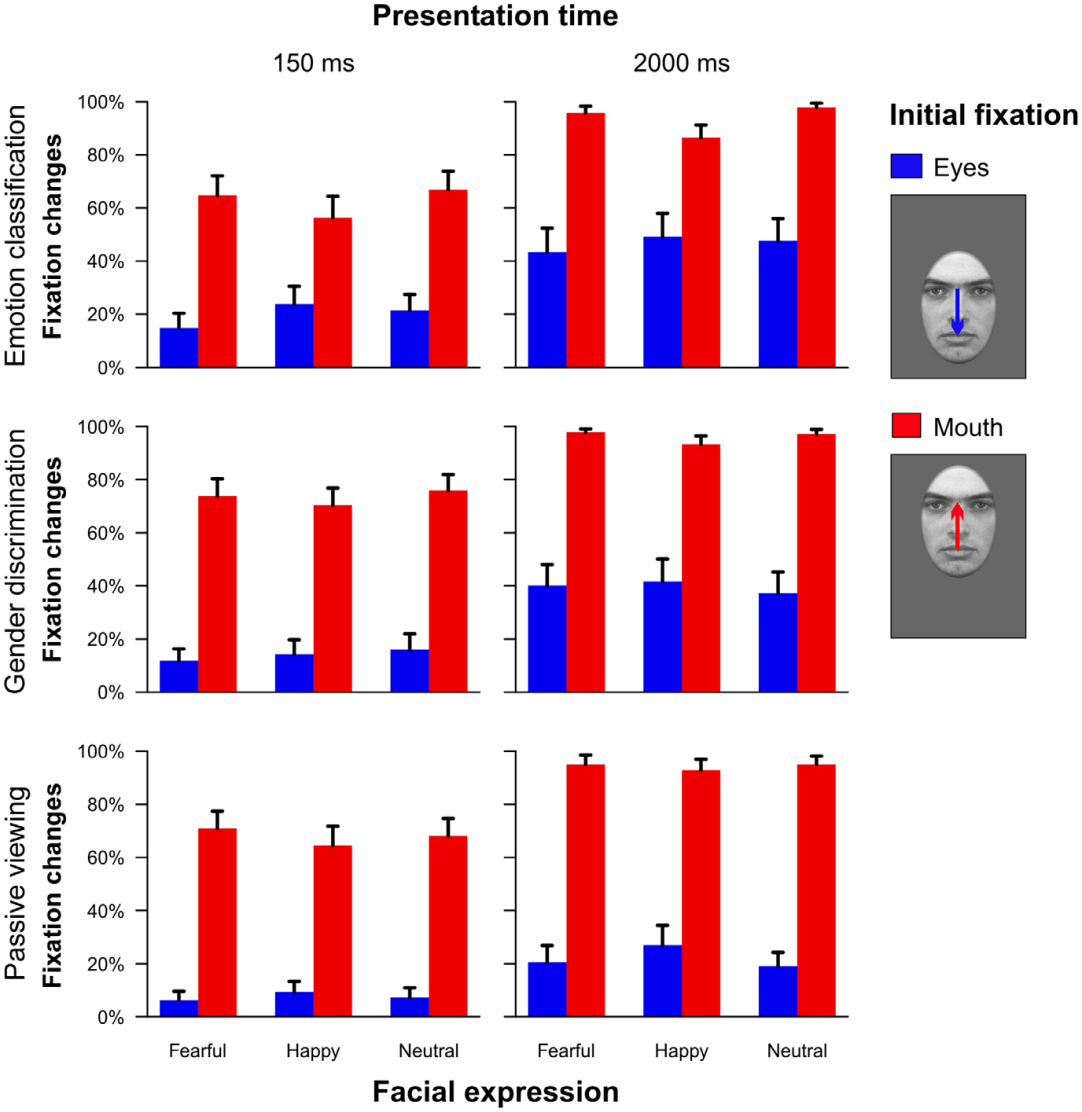
Proportions of fixation changes towards the other major facial feature as a function of task, presentation time, facial expression and initial fixation (Experiment 1). Error bars indicate standard errors of the mean.

Overall proportions of fixation changes as a function of task, emotional expression and initially fixated feature are depicted in Figure 3. It can be clearly seen that across all conditions there were far fewer saccades leaving the eye region than comparable fixation changes towards the eyes (main effect initial fixation: *F*_(1,23)_ = 83.80, *p*<.001, partial η^2^ = .79). Moreover, the proportion of fixation changes was larger for the longer presentation time (main effect presentation time: *F*_(1,23)_ = 40.05, *p*<.001, partial η^2^ = .64) and more saccades occurred in the emotion classification and the gender discrimination task than in the passive viewing condition (main effect task: *F*_(2,46)_ = 6.56, ε = .95, *p*<.01, partial η^2^ = .22). The proportion of fixation changes across different facial expressions were comparable and the main effect of emotion did not reach statistical significance (*F*_(2,46)_ = 0.84, ε = 1.00, *p* = .44, partial η^2^ = .04).

With respect to the interaction effects, we observed a statistically significant interaction between task and initial fixation (*F*_(2,46)_ = 8.40, ε = .90, *p*<.01, partial η^2^ = .27). If subjects initially fixated on the eyes, fixation changes occurred most often in the emotion classification task and least often in the passive task; but if they initially fixated on the mouth, the proportion of saccades was more similar across tasks (Figure 3).

**Figure 4:**
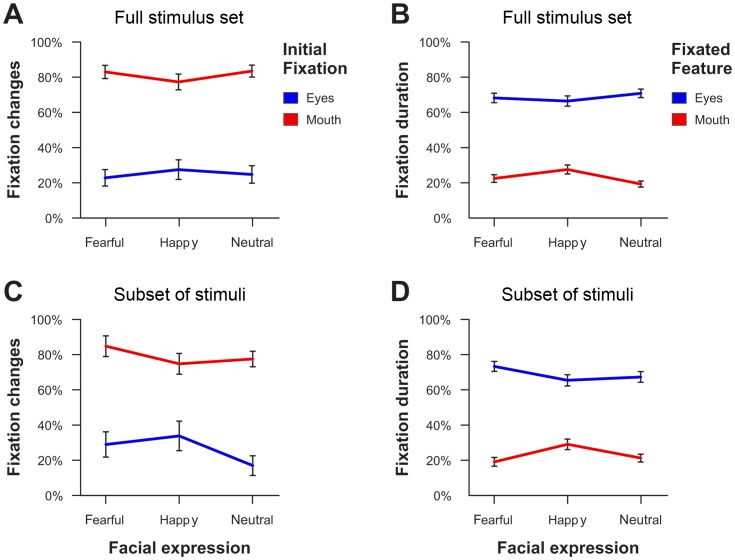
Illustration of the modulatory effect of facial expression on fixation changes (A,C) and fixation durations (B,D) across all experimental tasks and presentation times (Experiment 1). The upper panels (A,B) show the values for the whole stimulus set whereas the lower panels (C,D) only depict the respective values for a subset of faces with a comparable saliency ratio in the eye as compared to the mouth region. Error bars indicate standard errors of the mean.

Additionally, we observed an interaction between the factors emotion and initial fixation (*F*_(2,46)_ = 7.17, ε = 1.00, *p*<.01, partial η^2^ = .24; Figure 4A). When displaying fearful or neutral as opposed to happy facial expressions, participants showed a higher preference for the eye region. Thus, they tended to shift their gaze when initially looking at the mouth and they showed a lower number of saccades in the opposite direction. By contrast, participants showed a higher preference for the mouth region when viewing happy faces. This interaction effect was similar across tasks and presentation times (non-significant interaction task × emotion × initial fixation: *F*_(4,92)_ = 1.10, ε = .99, *p* = .36, partial η^2^ = .05; non-significant interaction presentation time × emotion × initial fixation: *F*_(2,46)_ = 0.52, ε = 1.00, *p* = .60, partial η^2^ = .02).

#### Eye tracking data: Fixation duration during the long presentation time (2000 ms)

**Figure 5:**
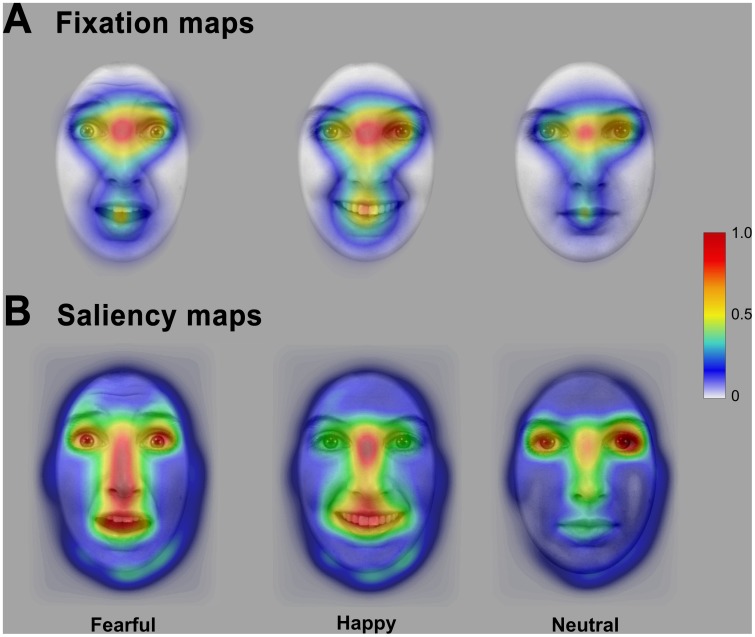
Heat maps illustrating the normalized fixation time on different face regions for the long presentation time of Experiment 1 (A) and the normalized distribution of saliency (B) as derived from a computational model of bottom-up visual attention [**13**], [**14**] for fearful, happy and neutral facial expressions.

Figure 5A illustrates the normalized cumulative time subjects spent looking at different regions of fearful, happy, and neutral faces. The overall density of fixations was higher when an emotional face (fearful or happy) was shown but across all expressions, participants fixated the eyes for a large amount of time. Additionally, the mouth of happy expressions was fixated longer than that of fearful or neutral faces. Note that the high density of fixations on the bridge of the nose most likely resulted from the initial fixation on that position in one half of all trials.

**Figure 6:**
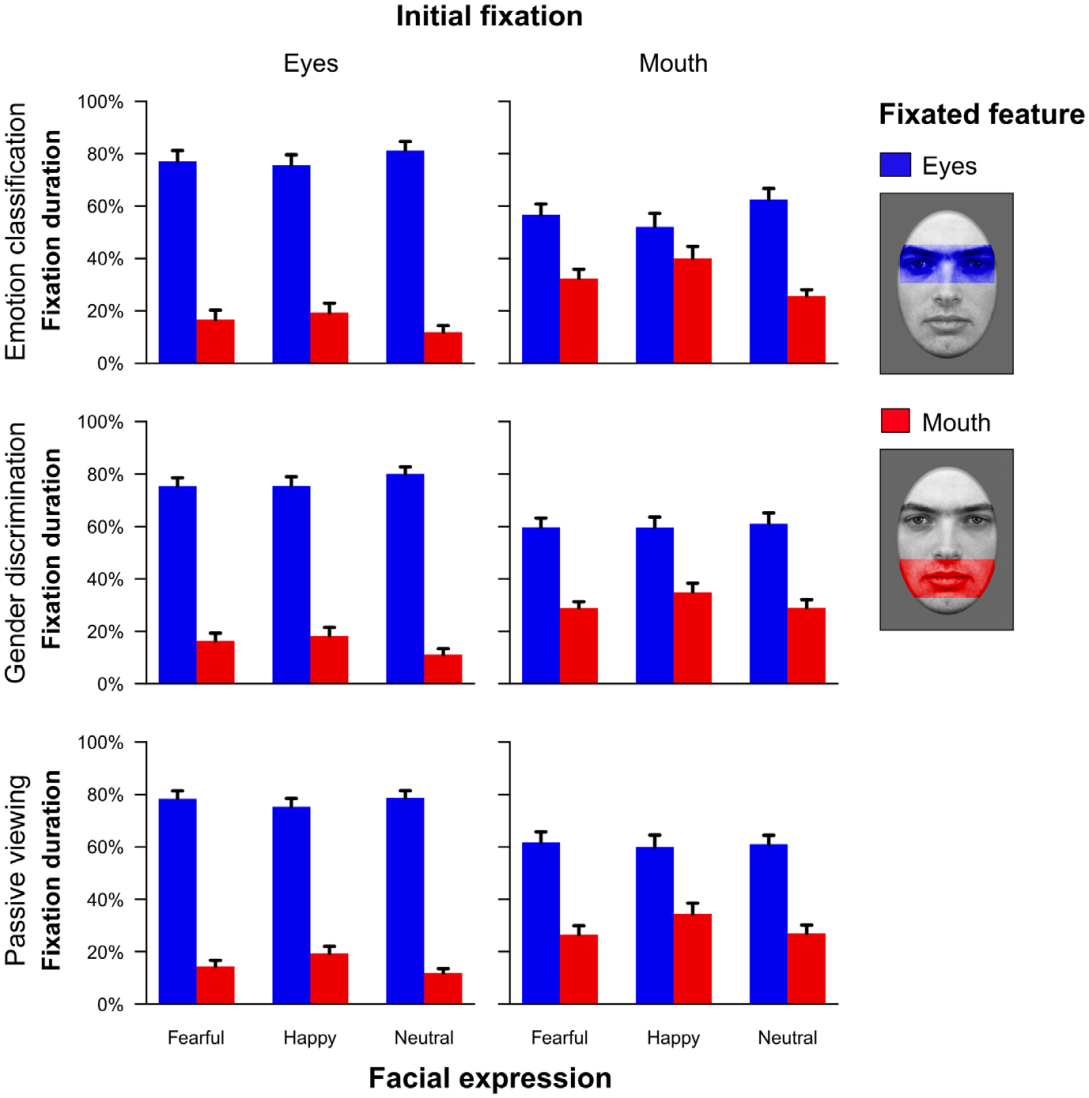
Proportion of time spent fixating either the eye or the mouth region in relation to the time subjects spent fixating the overall face in the long presentation time condition (Experiment 1). Mean proportions for fixations on the eye or mouth region are shown as a function of task, initial fixation and facial expression with error bars indicating standard errors of the mean. The regions of interest that were used to define fixations in the eye or mouth region, respectively, are shown on the right side.

For a more detailed analysis of the fixation data, proportions of time looking at either the eyes or the mouth relative to the whole time participants spent fixating the presented face were calculated. Because of missing data, results are based on 23 of the 24 subjects. Proportions of time spent fixating on the two major facial features as a function of task, initially fixated location and emotional expression can be seen in Figure 6. Overall, subjects fixated much longer on the eyes than on the mouth (main effect feature: *F*_(1,22)_ = 93.15, *p*<.001, partial η^2^ = .81). For happy facial expressions (main effect emotion: *F*_(2,44)_ = 17.10, ε = 1.00, *p*<.001, partial η^2^ = .44) as well as when initially looking at the eyes (main effect initial fixation: *F*_(1,22)_ = 7.33, *p*<.05, partial η^2^ = .25), the overall proportion of fixations on the eye or mouth region was slightly larger. All these effects were independent of the task at hand. Neither the main effect of task (*F*_(2,44)_ = 0.24, ε = 1.00, *p* = .79, partial η^2^ = .01) nor the interactions with the other three factors reached statistical significance.

Furthermore, we observed that the proportion of fixations on the eye or mouth region depended on the initial fixation (interaction between the factors initial fixation and fixated facial feature: *F*_(1,22)_ = 24.43, *p*<.001, partial η^2^ = .53). That is, when subjects initially fixated the eyes, they also showed longer fixations on the eye region across the whole trial. A similar pattern was observed when initially looking at the mouth (see Figure 6).

Corresponding to the saccadic data, subjects spent a larger amount of time fixating on the eyes when neutral or fearful faces were displayed but fixated the mouth more often when faces displayed a happy emotional expression (interaction between fixated facial feature and emotion: *F*_(2,44)_ = 19.34, ε = .86, *p*<.001, partial η^2^ = .47, see Figure 4B). Similar to the saccadic data, this effect was independent of the currently accomplished task (non-significant interaction task × emotion × fixated facial feature: *F*_(4,88)_ = 2.05, ε = .68, *p* = .12, partial η^2^ = .09).

#### Eye tracking data: The influence of low-level attentional saliency

Figure 5B shows the average saliency maps for all facial expressions. These maps indicate that the distribution of low-level stimulus characteristics that may drive eye movements differs between facial expressions. To examine whether this differential distribution can account for the observed pattern of saccades and fixations, we repeated the analyses for a subset of facial expressions that had a similar ratio of saliency in the eye and mouth region, respectively. Because of missing data, these analyses are based on 23 of the 24 subjects.

The analysis of the saccades that were triggered by the faces across tasks and presentation times revealed a significant main effect of initial fixation (*F*_(1,22)_ = 53.19, *p*<.001, partial η^2^ = .71) as well as a significant interaction of emotional expression and initial fixation (*F*_(2,44)_ = 3.43, ε = .87, *p*<.05, partial η^2^ = .13). As these effects are comparable to the original analysis for the whole stimulus set (see Figure 4C vs. 4A), they indicate that the distribution of low-level attentional features as a function of the emotional expression does not account for the observed effects.

A comparable analysis of the fixation data for the long presentation time (2000 ms) revealed significant main effects of facial feature (*F*_(1,22)_ = 106.56, *p*<.001, partial η^2^ = .83) and emotional expression (*F*_(2,44)_ = 5.50, ε = .93, *p*<.01, partial η^2^ = .20) as well as a significant interaction between both factors (*F*_(2,44)_ = 5.52, ε = 1.00, *p*<.01, partial η^2^ = .20). Comparable to the saccadic data, subjects spent more time fixating the eye region for fearful and neutral faces whereas a relatively enhanced fixation time was observed for the mouth region of happy facial expressions. As this pattern of results was also highly similar to the analysis of the whole stimulus set (see Figure 4D vs. 4B), it indicates that differences in the viewing pattern as a function of the emotional expression cannot be accounted for by low-level attentional processes.

Taken together, these results show that participants preferentially examine the eye region and show a bias to quickly scan diagnostic features of emotional facial expressions. That is, the eye region was more extensively scanned for fearful and neutral facial expressions whereas relatively more attention was paid to the mouth region when happy faces were shown. This effect is not related to differences in low-level image features since a post-hoc analysis of faces with a comparable ratio of saliency in the eye as compared to the mouth region revealed similar results as the analysis of the whole stimulus set. The general preference for scanning the eye region, however, might be a result of the larger amount of saliency in this image region. Moreover, since we manipulated the initial fixation on the faces, this general preference might also be related to the fact that the eyes were presented in the upper part of the visual field in one half of all trials whereas the mouth was never presented above the center of the screen. Thus, the supposed preference for the eye region can in principle result from a general bias to pay more attention to the upper part of the visual field. This bias might result from a different distribution of functional units in the lower and upper visual field [20], [21] and such bias was already demonstrated for eye movements in a visual search task [22]. To examine to what degree such bias is reflected in the results of this experiment, we carried out a second experiment where we manipulated the position where the faces were presented. Fearful, happy and neutral faces were either shown in the upper half, the middle or the lower half of the display screen. Moreover, stimuli were preselected according to the distribution of low-level image saliency in the eye and mouth region and we specifically selected a subset of faces with similar values in these facial areas to test whether participants would still show a preferential scanning of the eye region.

## Experiment 2

### Materials and Methods

#### Participants

This study was approved by the ethics committee of the medical association of Hamburg and conducted according to the principles expressed in the Declaration of Helsinki. All participants gave written informed consent and were paid for participation. Twenty-four subjects (12 women, 12 men) participated voluntarily in the experiment. They were aged between 22 and 41 years (*M* = 27.42; *SD* = 4.92 years). All had normal or corrected to normal vision and were informed beforehand to wear contact lenses instead of glasses when possible and to refrain from using eye make-up.

#### Design

The experiment was based on a 3 × 3 within-subjects design with the factors vertical placement and emotional expression. Faces were either presented in the upper half, in the middle or the lower half of the display screen. The horizontal displacement was varied randomly from trail to trial. Faces either showed fearful, happy, or neutral expressions.

#### Stimuli and tasks

The same stimulation and eye tracking equipment as in Experiment 1 was used. Stimuli were selected from the same pool of faces as in Experiment 1 according to the following criteria: One set of faces (24 for each emotional expression, respectively) had an equal ratio of saliency in the eye as compared to the mouth region. Mean values were *M* = 1.38 (*SD* = 0.07) for fearful, *M* = 1.37 (*SD* = 0.12) for happy, and *M* = 1.40 (*SD* = 0.11) for neutral facial expressions. These values did not differ significantly, *F*_(2,69)_ = 0.44, *p* = .65, partial η^2^ = .01. For the second stimulus set, we selected 12 fearful (saliency ratio *M* = 1.10, *SD* = 0.17) and 12 happy faces (*M* = 1.03, *SD* = 0.12) with a relatively similar saliency in the eye and mouth region that did not differ significantly between facial expressions, *t*(22) = 1.26, *p* = .22, partial η^2^ = .07. Such faces were not available for neutral facial expressions (see Figure 2) but we added 12 neutral faces with a higher saliency ratio (*M* = 1.76, *SD* = 0.14) to the stimulus set to avoid an overall reduced number of neutral faces. Eye tracking and behavioral data for these neutral faces were excluded from data analysis. Both stimulus sets consisted of an equal number of different male and female persons.

In the experimental task, faces of both stimulus sets were presented twice in a randomized order. Within each stimulus set, an equal number of faces was either presented in the upper half, the middle, or the lower half of the display screen by vertically shifting the center of the face (midpoint between the eye and mouth region) by 300, 0, or −300 pixels. The horizontal displacement was determined from trial to trial by drawing a random value from a uniform distribution ranging from −400 to 400 pixels. Participants were instructed to classify the facial expression as accurately and as quickly as possible by pressing the corresponding key on the computer keyboard.

Each trial began with a fixation cross shown for 1000 ms on a uniform grey background. Afterwards, faces were presented for 2000 ms followed by a 1000 ms blank screen. A fixation cross was again presented in the period between two successive trials that varied randomly between 1000 and 3000 ms. The total number of stimuli amounted to 216 faces (144 for stimulus set 1, 48 for set 2, 24 additional neutral faces) which were assigned to 3 sessions with short breaks in between to allow for a rest period and a recalibration of the eye tracker.

#### Procedure

The general procedure and the instructions for measuring eye movements were comparable to Experiment 1. Before starting the experimental task, 9 training trials were accomplished using a set of different faces. Before each session, the eye tracking system was calibrated using nine points and the calibration was validated.

#### Data reduction and analysis

All analyses were carried out separately for stimulus set 1 (equal ratio of saliency in the eye as compared to the mouth region across emotional expressions) and set 2 (similar saliency in the eye and mouth region of fearful and happy facial expressions). From the behavioral data, we calculated the proportion of correct emotion classifications and we examined effects of the experimental manipulations on the behavioral data using a 3 × 3 (stimulus set 1) or a 3 × 2 repeated measures ANOVA (stimulus set 2) with the factors vertical placement and emotional expression.

Comparable to Experiment 1, two different measures were extracted from the eye tracking data: First, we analyzed the first saccade that was accomplished after stimulus onset. Similar trial exclusion criteria as well as drift corrections as in Experiment 1 were used. Afterwards, the first saccade exceeding 1° within 2000 ms (i.e. during stimulus presentation) was detected. Furthermore, the saccade was required to occur at least 150 ms after stimulus onset. This saccade was classified according to whether it landed on the eye or the mouth region, respectively. For this purpose, the same regions of interest as in Experiment 1 were used. Finally, these numbers were divided by the total number of valid trials to obtain proportions of fixation changes as a function of the experimental manipulations. Using a 3 × 3 × 2 (stimulus set 1) or a 3 × 2 × 2 repeated measures ANOVA (stimulus set 2) on these proportions, we tested for effects of vertical placement, emotion and the facial feature where the first saccade landed (eye vs. mouth region).

Second, fixation durations were analyzed for the whole stimulus duration (2000 ms) using the same procedures as in Experiment 1. Valid trials were identified similarly to the saccadic data as described above. The cumulative fixation time on the eye and the mouth region was divided by the amount of time subjects spent looking at the presented face in general. To determine whether the experimental manipulations affected fixations on the eye and mouth region, we calculated a 3 × 3 × 2 (stimulus set 1) or a 3 × 2 × 2 repeated measures ANOVA (stimulus set 2) on these proportions using the factors vertical placement, emotion and the facial feature that was actually fixated.

For all repeated-measures ANOVAs involving more than one degree of freedom in the enumerator, the Huynh-Feldt procedure was applied to correct for potential violations of the sphericity assumption. A rejection region of *p*<.05 was used for all statistical tests and partial η^2^ is reported as an effect size index.

### Results and Discussion

#### Behavioral data

Similar to Experiment 1, participants were very accurate in the emotion classification task reaching mean proportions of correct responses above 95% in all experimental conditions (see Table 2). However, for stimulus set 1, participants were slightly less accurate in classifying fearful facial expressions (main effect emotion: *F*_(2,46)_ = 5.71, *p*<.01, partial η^2^ = .20). All other effects did not reach statistical significance and no statistically significant result was obtained for the behavioral data of the second stimulus set.

**Table 2:** Experiment 2: Proportions of correct responses in the emotion classification task as a function of vertical placement and emotional expression.

Vertical placement	Emotion		
	Fearful	Happy	Neutral
*Stimulus set 1*			
Upper screen half	95.3% (1.4%)	98.2% (0.7%)	98.4% (0.6%)
Middle of the screen	96.6% (0.8%)	97.7% (0.8%)	97.9% (0.8%)
Lower screen half	95.1% (1.1%)	96.9% (1.1%)	98.2% (0.8%)
*Stimulus set 2*			
Upper screen half	98.4% (0.9%)	97.4% (1.3%)	
Middle of the screen	98.4% (1.1%)	97.4% (1.1%)	
Lower screen half	98.4% (0.9%)	96.9% (1.4%)	

*Note*. Standard errors of the mean are printed in brackets.

#### Eye tracking data: First saccade after stimulus onset

The average number of valid trials without blinks or fixation changes between −300 and 150 ms relative to stimulus onset was 109.96 of 144 (*SD* = 24.17) for stimulus set 1 and 37.08 of 48 (*SD* = 9.00) for stimulus set 2. Since no valid trial was available for two conditions of stimulus set 2 for one participant, results on this stimulus set are based on 23 of the 24 subjects.

**Figure 7:**
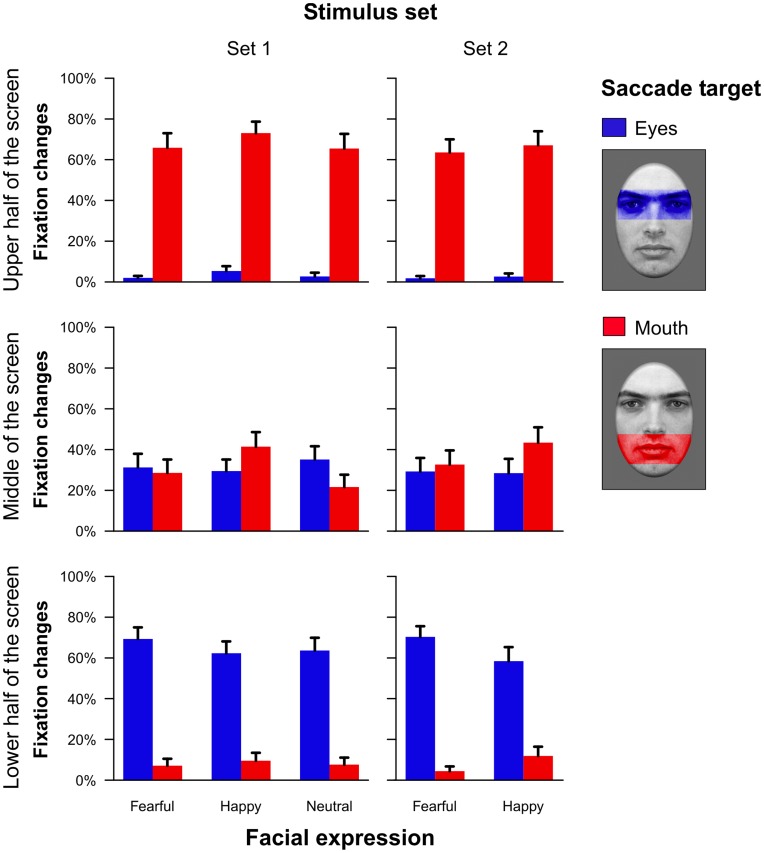
Proportions of fixation changes towards the eye and the mouth region as a function of the position in the visual field and the facial expression (Experiment 2). Results are depicted separately for a stimulus set with a similar saliency ratio of the eye as compared to the mouth region across facial expressions (stimulus set 1) and for a stimulus set with a saliency ratio of approximately 1 (stimulus set 2). The regions of interest that were used to define whether saccades targeted the eye or mouth region, respectively, are shown on the right side. Error bars indicate standard errors of the mean.

As depicted in Figure 7, proportions of saccades toward the eye or mouth region were highly similar across both stimulus sets. The first saccade after stimulus onset landed most frequently on the facial feature with the shortest spatial distance to the fixation cross that participants fixated at trial start. Thus, when the faces were presented in the upper half of the screen, participants tended to fixate the mouth region first whereas they showed more saccades to the eye region when faces were presented in the lower part of the screen. Consequently, a significant interaction between vertical placement and target region of the first saccade was obtained for stimulus set 1 (*F*_(2,46)_ = 74.83, ε = .98, *p*<.001, partial η^2^ = .76) as well as for set 2 (*F*_(2,44)_ = 59.96, ε = 1.00, *p*<.001, partial η^2^ = .73).

More interestingly, we additionally observed an interaction between the factors emotion and target region of the first saccade after stimulus onset for stimulus set 1 (*F*_(2,46)_ = 8.68, ε = 1.00, *p*<.001, partial η^2^ = .27) as well as for set 2 (*F*_(1,22)_ = 5.74, *p*<.05, partial η^2^ = .21). This effect replicates the results of Experiment 1 by revealing an enhanced proportion of saccades towards the eye as compared to the mouth region for fearful and neutral faces, whereas happy facial expressions triggered relatively more saccades toward the mouth. For stimulus set 1, this effect was slightly reduced when faces were presented in the upper part of the display screen (interaction of vertical placement, emotion and target of the saccade: *F*_(4,92)_ = 4.40, ε = 1.00, *p*<.01, partial η^2^ = .16). However, no such interaction was observed for stimulus set 2 (*F*_(2,44)_ = 1.50, ε = .87, *p* = .24, partial η^2^ = .06). Finally, a main effect of emotional expression was observed for stimulus set 1 (*F*_(2,46)_ = 6.62, ε = .93, *p*<.01, partial η^2^ = .22) indicating that across all other factors, slightly more saccades landed on the eye or mouth region when happy facial expressions were presented.

#### Eye tracking data: Fixation duration

**Figure 8:**
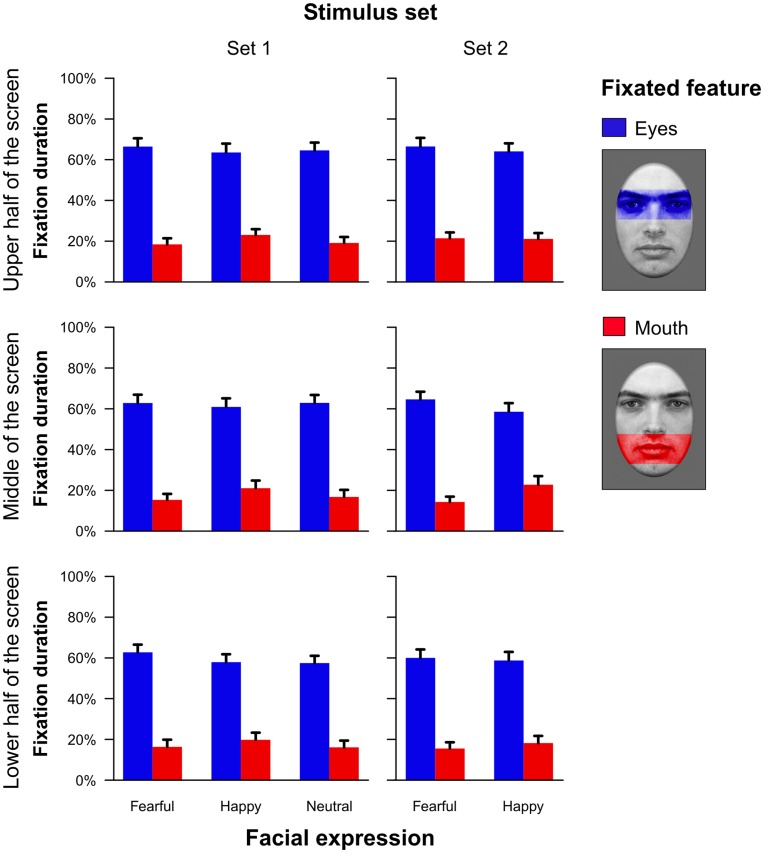
Proportion of time spent fixating either the eye or the mouth region in relation to the time subjects spent fixating the overall face (Experiment 2). Mean proportions for fixations on the eye or mouth region are shown as a function of the position in the visual field and the facial expression. Results are depicted separately for a stimulus set with a similar saliency ratio of the eye as compared to the mouth region across facial expressions (stimulus set 1) and for a stimulus set with a saliency ratio of approximately 1 (stimulus set 2). The regions of interest that were used to define fixations in the eye or mouth region, respectively, are shown on the right side. Error bars indicate standard errors of the mean.

Interestingly, the fixation durations showed a very different pattern as compared to the saccadic data. As shown in Figure 8, results were again highly similar for both stimulus sets. Participants spent much more time fixating the eye region as compared to the mouth in stimulus set 1 (*F*_(1,23)_ = 47.66, *p*<.001, partial η^2^ = .67) as well as set 2 (*F*_(1,22)_ = 54.14, *p*<.001, partial η^2^ = .71). This effect was similar for the different screen positions where the faces appeared. However, for both stimulus sets, participants showed a general bias to fixate less on the eye or mouth region when faces were shown in the lower part of the screen (main effect vertical placement in stimulus set 1, *F*_(2,46)_ = 6.34, ε = 1.00, *p*<.01, partial η^2^ = .22; and stimulus set 2, *F*_(2,44)_ = 6.67, ε = 1.00, *p*<.01, partial η^2^ = .23).

Most interestingly, and corresponding to the saccadic data, we again observed a significant interaction between emotion and fixated facial feature for stimulus set 1 (*F*_(2,46)_ = 8.84, ε = .79, *p*<.01, partial η^2^ = .28) and set 2 (*F*_(1,22)_ = 4.93, *p*<.05, partial η^2^ = .18). Similar to Experiment 1, participants spent a larger amount of time fixating on the eye as compared to the mouth region when neutral or fearful faces were displayed but fixated the mouth relatively longer when faces displayed a happy emotional expression. This effect was similar across the different vertical placements and no significant triple interactions were observed. Finally, the interaction between vertical placement and emotional expression was significant for stimulus set 1 (*F*_(4,92)_ = 2.76, ε = .96, *p*<.05, partial η^2^ = .11) indicating overall reduced fixations on the eye or mouth region of happy and neutral facial expressions when they were presented in the lower half of the display screen. All other effects did not reach statistical significance.

## Discussion

This study aimed at examining whether facial features that are diagnostic of the current emotional expression are automatically processed irrespective of the task at hand and the position in the visual field. In Experiment 1, eye movements were recorded while participants accomplished an emotion classification, a gender discrimination or a passive (oddball) task. To examine whether facial expressions trigger fast, potentially reflexive, eye movements, half of all stimuli were presented briefly (150 ms) whereas the other faces were shown for 2000 ms. The initial fixation was controlled for by unpredictably shifting faces either down- or upwards on each trial such that participants initially fixated on the eye or mouth region, respectively. In Experiment 2, participants were instructed to classify emotional facial expressions of faces that were presented at different positions in the upper half, the middle or the lower half of the display screen.

In both experiments, the amount of time spent looking at the eye region distinctly exceeded the amount of time spent looking at the mouth. Moreover, subjects in Experiment 1 accomplished few saccades leaving the eye region but shifted their gaze from the mouth to the eyes very often. This gazing pattern occurred even when saccades did not allow for extracting further information from the stimuli (i.e., for short presentation durations). Experiment 2 indicates that these results do not reflect a general bias for attending the upper visual field [22] but suggest that initial saccades target the facial feature that is nearest to fixation. These findings substantiate the conclusion that the eyes of a conspecific – even independent of the depicted emotional expression – provide important information that needs to be assessed quickly [23], [24]. Eye gaze is a crucial part of human non-verbal communication and interaction, as gaze cues signal the current focus of attention to the opponent and facilitate interactions [25]. In line with this reasoning, healthy but also autistic subjects begin exploring depicted faces significantly more often on the right or left eye than anywhere else [8]. These data suggest that the eyes play a crucial role in exploring and recognizing faces as well as in analyzing the emotional content of a particular expression. Our study further indicates that the eye region of conspecifics is processed preferentially across different facial expressions, positions in the visual field and experimental tasks.

Our second main finding was that the amount of fixation changes towards the eyes depended on the amount of diagnostic relevance of this facial feature for decoding the emotional expression [6]. This finding was stable across all tasks, presentation times and positions in the visual field. Subjects showed a higher preference for the eyes when viewing fearful or neutral expressions but they tended to shift their gaze more often towards the mouth when a happy face was presented. Similar effects were also obtained for fixation durations in both experiments: subjects spent more time looking at the eyes of neutral or fearful faces and fixated relatively longer on the mouth region when happy expressions were shown. This indicates that an evaluation of diagnostic emotional information takes place irrespective of experimental conditions. As we could find a dependency of gaze shifting on the particular diagnostic facial feature even when faces were shown very briefly in Experiment 1, this might imply that the processing of emotional expressions takes place preattentively [26], [27]. Such a preattentive emotion processing mechanism driving subsequent eye movements might be relevant for quickly and accurately determining another person’s emotional state. In human interactions, it is always useful to permanently know about an opponent’s feelings and intentions even if the current situation (i.e. “task”) does not require such information, because the interpersonal interaction might quickly shift to a topic that could require a precise assessment of the opponent’s feelings and intentions.

Additional analyses using a computational model of bottom-up visual attention [13], [14] indicate that both above-mentioned results, the preferential scanning of the eye region as well as the enhanced processing of diagnostically relevant facial features, could not be explained by low-level image properties. A reanalysis of the data of Experiment 1 only for stimuli with a comparable saliency ratio in the eye compared to the mouth region revealed results highly similar to the original analysis. Furthermore, comparable results were found with the specifically tailored stimulus set that was used in Experiment 2. Remarkably, participants fixated the eye region 3 to 4 times longer than the mouth region even for faces with a similar saliency in both parts of the face (stimulus set 2).

Interestingly, participants performed at ceiling in the experimental tasks of both experiments. Thus, even when the faces were only shown for 150 ms (Experiment 1) and subjects were unable to change their gaze from the initial fixation to a different facial feature, they were almost perfect in determining the gender or the emotional expression. This finding indicates that participants were able to perceive the face as a whole (which is also a necessary precondition for showing subsequent saccades toward specific diagnostic features) within this short time duration. Since we additionally refrained from using backward masking, face processing could continue even after stimulus offset. The observed early recognition of emotional expressions is in line with studies showing high face recognition rates after presentation times well below 200 ms [28] as well as larger amplitudes of early event-related brain potentials (N170) for facial displays containing diagnostic as compared to anti-diagnostic features [29]. Taken together, emotional expressions seem to be detected very early even without redirecting the gaze toward diagnostic features [30]. However, the observed pronounced tendency to shift the gaze to facial features that are diagnostic for the depicted emotional expression, which was unaffected by task demands and partly occurred after stimulus offset, indicates that attention is automatically shifted to such diagnostic features even when these saccades do not contribute to explicit emotion recognition.

To further characterize to what degree the observed effects rely on feature as compared to configural or holistic processing, it might be worthwhile to conduct future experiments with upright and inverted faces. In general, holistic processing seems to contribute to face perception since inverted faces are usually recognized slower than upright faces or other inverted objects [31]. However, this so-called face-inversion effect was found to be substantially reduced when directing participants’ gaze toward the eye region [32]. Moreover, face-inversion does not generally impair emotion recognition [33], [34], which indicates that even for inverted faces, a similar pattern of gaze changes as in the current study might be obtained.

With respect to the neurobiological underpinnings of the currently observed results, recent studies suggest that differential attention to facial features and specifically the eye region may be mediated by the amygdala. For example, it was shown that amygdala damage impairs spontaneous gaze fixation on the eye region of facial stimuli presented in an experimental setting [35] but also during free conversations [36]. We recently demonstrated that amygdala activation in healthy individuals predicted the amount of gaze changes toward the eye region of fearful faces [11]. Furthermore, a strong positive correlation between fusiform gyrus as well as amygdala activation and time spent fixating the eyes of happy, fearful, angry and neutral faces was shown in autistic subjects [37]. Consequently, the amygdala can be robustly activated by a variety of facial expressions [38] but activations seem to be largest when the eye region is crucial for determining the emotional state of the opponent (e.g., for fearful facial expressions [39]–[41]). These findings indicate that the strong preference for processing the eye region of others that was also found in the current study might rely on an involvement of the amygdala in detecting salient facial features in the visual periphery and directing attention toward them [42]. It remains an interesting question for future research to determine whether the relatively enhanced attention to the mouth region of happy faces that was found in the current study also relies on amygdala functioning. Benuzzi and colleagues recently reported increased amygdala activity to whole faces as compared to parts of faces displaying neutral expressions and they suggested that the amygdala might be involved in orienting attention toward socially relevant facial parts [43]. Transferred to the current study, it might be reasoned that diagnostic emotional features constitute such relevant parts and therefore, it remains an intriguing possibility that the amygdala also mediates directed attention toward these features. Of course, this hypothesis needs to be tested by future studies utilizing neuroimaging methods, for example.

The current study revealed a very consistent pattern of preferentially scanning the eye region and attending to emotional diagnostic facial features across several experimental conditions. The robustness of these findings in healthy individuals suggests that an application of the current experimental paradigm might also be advantageous in patient groups. For example, it is still debated whether patients with autism spectrum disorders scan (emotional) faces differentially than healthy observers [7], [10]. Reduced attention to the eye region in these patients has been linked to amygdala hypoactivation [44] and the variability in fixating the eye region within this group was found to be correlated with amygdala activity [37]. Using a comparable emotion classification task as in Experiment 1, it was recently demonstrated that individuals with autism spectrum disorders show an enhanced avoidance of the eye region for briefly presented emotional expressions [45]. In addition, the experimental paradigm of Experiment 1 offers the unique possibility to examine the time course of social attention in such patients in more detail by differentiating fast, potentially reflexive, eye movements from the subsequent scanning behavior that presumably is under conscious control. Moreover, it remains an interesting idea for future research to determine whether the degree of bottom-up attentional capture in patients with autism spectrum disorders differs from that in healthy controls. The computation of saliency maps and the comparison of eye movements to these low-level image statistics might be a first step into this direction.

A second clinical condition that seems to show abnormal face scanning is social anxiety disorder. In free viewing conditions, these patients tend to avoid fixating the eye region of conspecifics [46]. Instead, they show hyperscanning as reflected by increased scanpath length and short fixation periods also on non-diagnostic features such as hair or ears [47]. Interestingly, these patients show hyperactivation of the amygdala [48]–[50]. In line with the above mentioned hypothesized functional role of the amygdala, one may speculate that patients with social phobia initially show enhanced reflexive gaze shifts toward the eye region but subsequently avoid scanning this feature to reduce an upcoming fear of being observed and evaluated by others. This hypothesis can be addressed by the experimental paradigm of Experiment 1. Patients with social phobia would be supposed to show a large number of initial gaze shifts toward the eye region but for a longer viewing duration, one would predict this pattern to change into enhanced attention to non-diagnostic features and an active avoidance of focusing on the eye region.

To sum up, our study clearly underlined the importance of an opponent’s eye region in driving gazing behavior. Moreover, we showed that diagnostic facial features of emotional expressions are preferentially processed irrespective of task demands, position in the visual field and low-level image statistics. These gaze preferences were evident very early after stimulus onset and occurred even when saccades did not allow for extracting further information from these stimuli. Thus, they may result from an automatic detection of salient features in the visual field.

## References

[1] Martino B De, Kalisch R, Rees G, Dolan RJ (2009). Enhanced processing of threat stimuli under limited attentional resources.. Cereb Cortex.

[2] Palermo R, Rhodes G (2007). Are you always on my mind? A review of how face perception and attention interact.. Neuropsychologia.

[3] Hanawalt NG (1944). The role of the upper and the lower parts of the face as a basis for judging facial expressions: II. In posed expressions and “candid-camera” pictures.. J Gen Psychol.

[4] Gosselin F, Schyns PG (2001). Bubbles: a technique to reveal the use of information in recognition tasks.. Vision Res.

[5] Schyns PG, Bonnar L, Gosselin F (2002). Show me the features! Understanding recognition from the use of visual information.. Psychol Sci.

[6] Smith ML, Cottrell GW, Gosselin F, Schyns PG (2005). Transmitting and decoding facial expressions.. Psychol Sci.

[7] Rutherford MD, Towns AM (2008). Scan path differences and similarities during emotion perception in those with and without autism spectrum disorders.. J Autism Dev Disord.

[8] Hernandez N, Metzger A, Magné R, Bonnet-Brilhault F, Roux S (2009). Exploration of core features of a human face by healthy and autistic adults analyzed by visual scanning.. Neuropsychologia.

[9] Pelphrey K a, Sasson NJ, Reznick JS, Paul G, Goldman BD (2002). Visual scanning of faces in autism.. J Autism Dev Disord.

[10] Spezio ML, Adolphs R, Hurley RSE, Piven J (2007). Abnormal use of facial information in high-functioning autism.. J Autism Dev Disord.

[11] Gamer M, Büchel C (2009). Amygdala activation predicts gaze toward fearful eyes.. J Neurosci.

[12] Vinette C, Gosselin F, Schyns PG (2004). Spatio-temporal dynamics of face recognition in a flash: it’s in the eyes.. Cognitive Sci.

[13] Itti L, Koch C (2000). A saliency-based search mechanism for overt and covert shifts of visual attention.. Vision Res.

[14] Itti L, Koch C (2001). Computational modelling of visual attention.. Nat Rev Neurosci.

[15] Lundqvist D, Flykt A, Öhman A (1998). The Karolinska Directed Emotional Faces - KDEF, CD ROM..

[16] Ekman P, Friesen WV (1976). Pictures of facial affect.. Palo Alto, CA: Consulting Psychologists Press.

[17] Ebner NC, Riediger M, Lindenberger U (2010). FACES - a database of facial expressions in young, middle-aged, and older women and men: development and validation.. Behav Res Meth.

[18] Gamer M, Zurowski B, Büchel C (2010). Different amygdala subregions mediate valence-related and attentional effects of oxytocin in humans.. Proc Natl Acad Sci U S A.

[19] Itti L, Koch C, Niebur E (1998). A model of saliency-based visual attention for rapid scene analysis.. IEEE Trans Pattern Anal Mach Intell.

[20] Previc FH, Blume L (1993). Visual search asymmetries in three-dimensional space.. Vision Res.

[21] Levine MW, McAnany JJ (2005). The relative capabilities of the upper and lower visual hemifields.. Vision Res.

[22] Pflugshaupt T, Wartburg R von, Wurtz P, Chaves S, Déruaz A (2009). Linking physiology with behaviour: Functional specialisation of the visual field is reflected in gaze patterns during visual search.. Vision Res.

[23] Emery N (2000). The eyes have it: the neuroethology, function and evolution of social gaze.. Neurosci Biobehav R.

[24] Langton S, Watt R, Bruce I (2000). Do the eyes have it? Cues to the direction of social attention.. Trends Cogn Sci.

[25] Baron-Cohen S, Moore C, Dunham PJ (1995). The eye direction detector (EDD) and the shared attention mechanism (SAM): Two cases for evolutionary psychology..

[26] Pizzagalli D, Regard M, Lehmann D (1999). Rapid emotional face processing in the human right and left brain hemispheres: an ERP study.. Neuroreport.

[27] White M (1995). Preattentive Analysis of Facial Expressions of Emotion.. Cogn Emot.

[28] Genetti M, Khateb A, Heinzer S, Michel CM, Pegna AJ (2009). Temporal dynamics of awareness for facial identity revealed with ERP.. Brain Cogn.

[29] Joyce C a, Schyns PG, Gosselin F, Cottrell GW, Rossion B (2006). Early selection of diagnostic facial information in the human visual cortex.. Vision Res.

[30] Eimer M, Holmes A (2007). Event-related brain potential correlates of emotional face processing.. Neuropsychologia.

[31] Valentine T (1988). Upside-down faces: A review of the effect of inversion upon face recognition.. Br J Psychol.

[32] Hills PJ, Ross D a, Lewis MB (2011). Attention misplaced: the role of diagnostic features in the face-inversion effect.. J Exp Psychol Hum Percept Perform.

[33] Lipp OV, Price SM, Tellegen CL (2009). No effect of inversion on attentional and affective processing of facial expressions.. Emotion.

[34] Arnold DH, Lipp OV (2011). Discrepant integration times for upright and inverted faces.. Perception.

[35] Adolphs R, Gosselin F, Buchanan TW, Tranel D, Schyns P (2005). A mechanism for impaired fear recognition after amygdala damage.. Nature.

[36] Spezio ML, Huang P-YS, Castelli F, Adolphs R (2007). Amygdala damage impairs eye contact during conversations with real people.. J Neurosci.

[37] Dalton KM, Nacewicz BM, Johnstone T, Schaefer HS, Gernsbacher MA (2005). Gaze fixation and the neural circuitry of face processing in autism.. Nat Neurosci.

[38] Fitzgerald D a, Angstadt M, Jelsone LM, Nathan PJ, Phan KL (2006). Beyond threat: amygdala reactivity across multiple expressions of facial affect.. NeuroImage.

[39] Morris JS, Friston KJ, Büchel C, Frith CD, Young AW (1998). A neuromodulatory role for the human amygdala in processing emotional facial expressions.. Brain.

[40] Morris JS, Frith CD, Perrett DI, Rowland D, Young AW (1996). A differential neural response in the human amygdala to fearful and happy facial expressions.. Nature.

[41] Whalen PJ, Kagan J, Cook RG, Davis FC, Kim H (2004). Human amygdala responsivity to masked fearful eye whites.. Science.

[42] Adolphs R (2008). Fear, faces, and the human amygdala.. Curr Opin Neurobiol.

[43] Benuzzi F, Pugnaghi M, Meletti S, Lui F, Serafini M (2007). Processing the socially relevant parts of faces.. Brain Res Bull.

[44] Baron-Cohen S, Ring HA, Bullmore ET, Wheelwright S, Ashwin C (2000). The amygdala theory of autism.. Neurosci Biobehav R.

[45] Kliemann D, Dziobek I, Hatri A, Steimke R, Heekeren HR (2010). Atypical Reflexive Gaze Patterns on Emotional Faces in Autism Spectrum Disorders.. J Neurosci.

[46] Horley K, Williams LM, Gonsalvez C, Gordon E (2003). Social phobics do not see eye to eye: a visual scanpath study of emotional expression processing.. J Anxiety Disord.

[47] Horley K, Williams LM, Gonsalvez C, Gordon E (2004). Face to face: visual scanpath evidence for abnormal processing of facial expressions in social phobia.. Psychiatry Res.

[48] Straube T, Kolassa I-T, Glauer M, Mentzel H-J, Miltner WHR (2004). Effect of task conditions on brain responses to threatening faces in social phobics: an event-related functional magnetic resonance imaging study.. Biol Psychiatry.

[49] Straube T, Mentzel H-J, Miltner WHR (2005). Common and distinct brain activation to threat and safety signals in social phobia.. Neuropsychobiology.

[50] Phan KL, Fitzgerald DA, Nathan PJ, Tancer ME (2006). Association between amygdala hyperactivity to harsh faces and severity of social anxiety in generalized social phobia.. Biol Psychiatry.

